# Annexins and autoantibodies in autoimmune diseases – Insights into SLE, APS and RA: A review

**DOI:** 10.17305/bb.2026.13546

**Published:** 2026-01-16

**Authors:** Xiuli Zhou, Jinle Liu, Siyi Wang, Yexiao Zhang, Linjie Xu, Lan Wu

**Affiliations:** 1 Department of Obstetrics, Women’s Hospital of Nanjing Medical University, Nanjing Women and Children’s Healthcare Hospital, Nanjing, Jiangsu, China

**Keywords:** Annexin family, autoantibodies, autoimmune diseases, systemic lupus erythematosus, antiphospholipid syndrome

## Abstract

Autoimmune diseases are becoming increasingly prevalent and can cause multi-organ damage through dysregulated immune responses to self-antigens. This review aims to summarize the roles of annexin family proteins and annexin autoantibodies in the mechanisms of autoimmune diseases, as well as their potential diagnostic and therapeutic applications. A targeted PubMed search conducted on August 31, 2025, utilized annexin- and disease-related terms without year restrictions, focusing on English-language, peer-reviewed studies involving humans or recognized animal models. Evidence suggests that Annexin A1 (ANXA1) and formyl peptide receptor 2 (FPR2) signaling can influence inflammatory and T-cell responses. Additionally, Annexin A2 (ANXA2) is associated with organ-targeted injury, such as lupus nephritis (LN) in systemic lupus erythematosus (SLE), through its interactions with anti-double-stranded DNA antibodies (anti-dsDNA). Annexin A5 (ANXA5) serves as an anticoagulant phospholipid “shield,” which can be compromised by antiphospholipid antibodies (aPLs), contributing to thrombosis and obstetric complications in antiphospholipid syndrome (APS) and increasing vascular risk in SLE. In rheumatoid arthritis (RA), ANXA1 exhibits context-dependent effects, while ANXA2 promotes synovial proliferation, invasion, and angiogenesis. Dysregulation of annexins has also been observed in primary Sjögren’s syndrome (pSS), multiple sclerosis (MS), and systemic sclerosis (SSc). Additionally, the emerging utility of anti-ANXA1, anti-ANXA2, and anti-ANXA5 autoantibodies for phenotyping and risk stratification, including in seronegative antiphospholipid syndrome (SNAPS), highlights their clinical relevance. Overall, annexins and their autoantibodies represent promising biomarkers and therapeutic targets; however, the heterogeneity of assays and the limited availability of prospective multicenter data currently hinder clinical translation.

## Introduction

Autoimmune diseases result from an overactive immune response to self-antigens, leading to tissue and organ damage. These conditions encompass a wide range of disorders, including systemic lupus erythematosus (SLE), antiphospholipid syndrome (APS), and rheumatoid arthritis (RA). Epidemiological studies estimate that approximately 5% of the global population is affected by these diseases, with incidence rates continuing to rise [1–3]. Therefore, in-depth investigations into their pathogenesis, the development of early diagnostic methods, and optimized therapeutic strategies are of paramount importance.

Annexins are a class of calcium-dependent phospholipid-binding proteins that play critical roles in biological processes such as vesicular trafficking, autophagy, inflammatory responses, and cell signaling [4, 5]. This protein family is implicated in various human diseases, including tumorigenesis, female reproductive disorders, obesity, and atherosclerosis [6–10]. Recent studies suggest that specific members of the annexin family and their autoantibodies are dysregulated in multiple autoimmune diseases, positioning them as potential diagnostic biomarkers and therapeutic targets. However, a systematic summary of the roles of annexins in autoimmune diseases is currently lacking. Consequently, this article aims to review the expression profiles and functional mechanisms of the annexin family in autoimmune diseases to provide a reference for related research.

To support this narrative review, a targeted literature search was conducted in the PubMed electronic database on August 31, 2025. The search strategy included the following primary Medical Subject Headings (MeSH) terms and related keywords: annexin, Annexin A1 (ANXA1), Annexin A2 (ANXA2), Annexin A5 (ANXA5), autoimmune disease, SLE, APS, RA, primary Sjögren’s syndrome (pSS), multiple sclerosis (MS), and systemic sclerosis (SSc). The search terms were combined using Boolean operators “AND” and “OR,” with no publication year restrictions, limited to English literature. Inclusion criteria were: (1) Study subjects: humans or recognized animal models of autoimmune diseases; (2) Study content: involvement of annexin family members or their autoantibodies in expression, function, or clinical significance; (3) Publication type: published in peer-reviewed academic journals.

## The annexin family

The annexin family comprises a group of calcium-dependent phospholipid-binding proteins encoded by multiple genes and conserved across eukaryotes. The human annexin family includes 12 members (A1–A11 and A13) [11, 12]. Their canonical structure consists of a conserved C-terminal core domain and a variable N-terminal domain. The core domain typically contains four repeats of approximately 70 amino acids each (annexin A6 contains eight repeats) and harbors characteristic type II Ca^2+^ binding sites, enabling specific recognition and binding to negatively charged phospholipids. The N-terminal domain exhibits significant length polymorphism and sequence diversity, featuring various post-translational modification sites crucial for regulating annexin function [5, 11, 13]. This family is implicated in vital processes such as plasma membrane dynamics, inflammation, coagulation, and apoptosis, with A1, A2, and A5 being the most extensively studied members (Table 1).

**Table 1:** Key features of annexin in autoimmune diseases

**Annexin**	**Amino** **acid count**	**Molecular** **weight (kDa)**	**Key functional sites/motifs in the** **N-terminal domain**	**Primary functions**
ANXA1	346	37	Tyr21/Ser27, Trp12/Lys26, QAWFI.	1. Dual Role in Inflammation. 2. Involvement in plasma membrane repair, cell cycle progression, and apoptos is regulation, among other processes.
ANXA2	339	38	Ser1, Ser11, Ser25, Tyr23, S100A10 binding site.	1. Intracellularly: Regulates endo-/exocytosis, multivesicular body formation, cell cycle and proliferation, apoptos is, inflammatory responses, and cell signal transduction. 2. Extracellularly: Participates in phagocytosis, promotes fibrinolysis, exerts anticoagulant functions, and facilitates angiogenesis.
ANXA5	320	35	Lacks canonical post-translational modification sites.	Involved in anticoagulation, cytoprotection, and inflammation regulation, among other processes.

Abbreviations: ANXA1: Annexin A1; ANXA2: Annexin A2; ANXA5: Annexin A5.

### ANXA1

ANXA1 is a 37 kDa protein composed of 346 amino acids, highly expressed in immune cells (neutrophils, monocytes, macrophages) and various tissues. Its N-terminal domain contains two α-helices arranged at a 60∘ tilt (Ala2-Asn16 and Glu18-Lys26) and features several functional sites: epidermal growth factor receptor (EGFR)/protein kinase C (PKC)-dependent phosphorylation sites (Tyr21/Ser27), cathepsin D/calpain I cleavage sites (Trp12/Lys26), and a peptide motif (QAWFI) mediating interaction with S100A11 [9, 14].

ANXA1 exhibits a unique dual role in inflammation. Its anti-inflammatory effects are primarily mediated by glucocorticoids (GCs) through binding to formyl peptide receptor 2 (FPR2) of the G-protein coupled receptor family, promoting neutrophil apoptosis and downregulating the expression of intracellular adhesion molecule (ICAM) and vascular cell adhesion molecule 1 (VCAM1). Under specific conditions (e.g., proteolytic cleavage), ANXA1 can be converted into a pro-inflammatory form. Furthermore, ANXA1 participates in other critical physiological processes, including plasma membrane repair, cell cycle progression, and apoptosis regulation [14–17].

### ANXA2

ANXA2 is a 38 kDa protein comprising 339 amino acids. Its N-terminal domain consists of 30 amino acid residues, an acetylation site (Ser1), three phosphorylation sites (Ser11, Ser25, and Tyr23), and an S100A10 binding site. ANXA2 exists as a monomer or a heterotetramer (A2t) in various cell types, including monocytes, endothelial cells, and myeloid cells. The monomer is primarily localized in the cytoplasm, with a minor nuclear fraction, whereas the heterotetramer, formed by two ANXA2 monomers bridged non-covalently by an S100A10 dimer, is typically located beneath the plasma membrane or within the cytoskeletal region [18, 19]. This structural diversity and subcellular compartmentalization underlie its multifunctional regulatory capacity.

ANXA2 exerts important biological functions both intracellularly and extracellularly. Intracellularly, it regulates endo-/exocytosis, mediates multivesicular body formation, modulates cell cycle and proliferation, and plays key roles in apoptosis and inflammatory responses. Notably, ANXA2 stabilizes lipid raft structures via interaction with CD44, thereby regulating cell signal transduction through the lipid raft-cytoskeleton axis. Extracellularly, ANXA2 participates in phagocytosis, promotes fibrinolysis, exerts anticoagulant functions, and plays a significant role in angiogenesis [18, 20, 21].

### ANXA5

ANXA5 is a single-chain protein with a molecular weight of approximately 35 kDa, consisting of 320 amino acids. It is the most abundant annexin isoform in nearly all cell types except neurons, primarily expressed by trophoblasts and vascular endothelial cells, and is also widely distributed in other cells, tissues, and the circulation [13, 22]. Unlike ANXA1 and A2, its N-terminal domain comprises approximately 20 amino acids and lacks canonical post-translational modification sites. This streamlined structure minimizes steric hindrance, allowing the core domain to bind phosphatidylserine (PS) with high affinity, a property fundamental to ANXA5’s roles in anticoagulation, cytoprotection, and inflammation regulation [23–25].

## The annexin family and SLE

SLE is a classic chronic systemic autoimmune disease characterized by inflammation and immune-mediated damage across multiple organ systems. Commonly affected organs include the skin and mucous membranes, kidneys, heart, lungs, and the hematopoietic system [26]. Globally, approximately 400,000 individuals are newly diagnosed with SLE each year, predominantly affecting women of childbearing age, with a female-to-male ratio of 9:1 [27]. The pathogenesis of SLE is believed to involve interactions among genetic predispositions, environmental factors, and immune dysregulation. These complex interactions lead to the production of pathogenic autoantibodies, which form immune complexes (ICs) upon binding to their corresponding antigens. In SLE, the accumulation of autoantibodies and ICs within various tissues and organs triggers inflammation, ultimately causing damage [28, 29]. Recent research indicates that ANXA1, ANXA2, and ANXA5 may play significant roles in the pathogenesis of SLE and its associated complications.

### ANXA1 and SLE

ANXA1 modulates T-cell receptor (TCR) signaling through its interaction with FPR2, thereby influencing the activation threshold of T-cells [30]. Studies have demonstrated that the ANXA1-FPR2 pathway contributes to T-cell activation and differentiation, playing a crucial role in the development of SLE [31]. Animal studies reveal that blocking the ANXA1-FPR2 pathway using an ANXA1-specific monoclonal antibody in SLE-prone mouse models suppresses disease symptoms and autoantibody production, subsequently prolonging survival [32]. A case-control study conducted in a Southern Tunisian population also identified associations between polymorphisms in ANXA1, FPR1, and FPR2 genes and susceptibility to SLE [33]. Collectively, these findings suggest a significant role for ANXA1 in the pathogenesis of SLE.

### ANXA2 and SLE

Lupus nephritis (LN) is one of the most severe and prevalent complications of SLE, representing a major risk factor for morbidity and mortality and potentially leading to end-stage renal disease (ESRD)[34]. Over 10% of renal biopsies are diagnosed as LN, affecting approximately 40% of SLE patients [35]. LN is characterized by the accumulation of autoantibodies, primarily anti-double-stranded DNA (anti-dsDNA), in the glomeruli and interstitium, which activates the complement system and recruits immune cells, initiating inflammation and subsequent organ damage [36]. However, the mechanisms by which autoantibodies localize to target organs and induce injury remain partially understood. It has been proposed that anti-dsDNA antibodies can bind directly to ANXA2 on mesangial cells, inducing downstream inflammatory processes [37]. Studies indicate that 65% of anti-dsDNA antibodies isolated from LN patients, as well as nearly all antibody samples obtained during disease flares, exhibit significant binding to ANXA2. Furthermore, human and murine LN biopsies reveal co-localization of ANXA2 with ICs in glomeruli, further supporting its pathological association with anti-dsDNA antibodies [38].

### ANXA5 and SLE

Cardiovascular disease (CVD) associated with SLE can be viewed as a late-stage complication and a significant contributor to the elevated mortality rate observed in SLE patients [39, 40]. Independent risk factors for CVD in this population include traditional factors such as age, dyslipidemia, hypertension, diabetes, and smoking, along with non-traditional factors including antiphospholipid antibodies (aPLs), other manifestations of SLE (especially renal), corticosteroid therapy, and low levels of natural antibodies] [41]. Approximately 30%–40% of SLE patients test positive for aPLs [42], which can promote thrombosis by disrupting the anticoagulant properties of ANXA5 [41]. Recent research has shown that ANXA5 typically binds to susceptible regions of atherosclerotic plaques to maintain their stability. Disruption of ANXA5 by aPLs increases the risk of plaque rupture, thereby elevating the likelihood of myocardial infarction (MI) and stroke in SLE patients [43]. Additionally, oxidized cardiolipin (oxCL), a product of cardiolipin oxidation, exacerbates endothelial dysfunction and thrombosis in SLE patients while synergizing with aPLs to promote CVD [44]. Importantly, ANXA5 can counteract this effect by binding to oxCL with high affinity through its phospholipid-binding domain, thus exerting a protective role [45, 46].

## The annexin family and APS

APS is a non-inflammatory autoimmune disorder characterized by the persistent presence of aPLs, with primary clinical manifestations including recurrent arterial and/or venous thrombosis and adverse pregnancy outcomes [47]. APS typically affects relatively young individuals and shows a female predominance. The estimated prevalence of APS in the population is 0.04%–0.05%, with an annual incidence of 0.001%–0.002% [48, 49]. aPLs are regarded as the primary mediators of APS pathogenesis, facilitating characteristic clinical manifestations such as thrombosis and obstetric complications through various pathological mechanisms. Notably, the annexin family, particularly ANXA2 and ANXA5, is recognized for its significant regulatory roles in APS clinical presentations.

### ANXA2 and APS

As a common acquired thrombophilia, APS can lead to recurrent arterial and venous thrombosis. ANXA2 functions as a receptor on the cell surface, facilitating the assembly of plasminogen and tissue-type plasminogen activator (t-PA), thereby significantly enhancing fibrinolytic capacity [50]. In APS patients, anti-ANXA2 antibodies inhibit this plasminogen-activating function by binding to ANXA2, disrupting the delicate balance of the coagulation-fibrinolysis system and leading to pathological thrombosis [51–53]. Furthermore, ANXA2 on the endothelial cell surface can bind to β2-glycoprotein I (β2GPI), forming a stable β2GPI-ANXA2 complex. In susceptible individuals, this complex may stimulate the production of anti-β2GPI antibodies (aβ2GPI), with cross-linking of aβ2GPI and β2GPI promoting endothelial cell activation and exacerbating thrombosis [52, 54].

### ANXA5 and APS

As previously mentioned, aPLs can disrupt the antithrombotic barrier function of ANXA5, thereby increasing the risk of thrombosis [41, 55, 56]. In APS patients, the interaction between anti-ANXA5 antibodies and ANXA5 further compromises this anticoagulant barrier, promoting thrombosis. Additionally, disruption of the ANXA5 anticoagulant shield on trophoblast cells by aPLs and anti-ANXA5 antibodies can induce placental thrombosis, leading to adverse pregnancy outcomes [52, 57]. Morbid pregnancy is the second most common clinical manifestation of APS, including recurrent spontaneous abortion (RSA), fetal growth restriction (FGR), preeclampsia (PE), and preterm birth [58]. Notably, the pathogenesis of ANXA5-related morbid pregnancy cannot be solely attributed to placental thrombosis. *In vitro* studies indicate that anti-ANXA5 antibodies can promote trophoblast apoptosis and significantly inhibit human chorionic gonadotropin (hCG) secretion by interfering with ANXA5’s physiological functions, contributing to adverse pregnancy outcomes [8, 59]. However, further investigation is required to elucidate the precise mechanisms of ANXA5 in APS-related obstetric complications.

## The annexin family and RA

RA is a chronic autoimmune disease pathologically characterized by persistent synovial hyperplasia, inflammatory cell infiltration, pannus formation, and cartilage and bone erosion. It typically manifests as symmetric polyarthritis, predominantly affecting the small joints of the extremities, such as the metacarpophalangeal and proximal interphalangeal joints. This condition can lead to joint deformity and functional impairment, impacting approximately 0.5%–1% of the global population. The pathogenesis of RA involves multifactorial interactions, including autoimmune dysregulation, inflammatory cascades, genetic predisposition, and metabolic reprogramming of synovial cells [60–62]. Notably, altered expression of annexin family members in RA patients suggests they may play critical roles in disease pathogenesis.

### ANXA1 and RA

ANXA1 exhibits a complex dual regulatory role in RA. As a key anti-inflammatory protein induced by GCs, ANXA1 suppresses excessive immune responses by modulating neutrophil and T cell functions and the FPR2 receptor signaling pathway [63]; Concurrently, it inhibits ANXA1–cytosolic phospholipase A2 (cPLA2α) activity, reducing the production of arachidonic acid and its pro-inflammatory metabolites, particularly prostaglandin E2 (PGE2) and leukotriene B4 (LTB4), while upregulating the anti-inflammatory cytokine IL-10, thereby alleviating synovial inflammation in RA [64, 65]. Animal studies further confirm that ANXA1 can improve RA-associated cardiac diastolic dysfunction [66]. However, recent research indicates that ANXA1 may promote disease progression under certain conditions; for instance, activation of the lncRNA-Anrel/miR-146a/ANXA1 axis exacerbates joint inflammation [67]. These findings underscore the context-dependent role of ANXA1 in RA, warranting further investigation.

### ANXA2 and RA

Research indicates that the expression and phosphorylation levels of ANXA2 are significantly elevated in the synovial tissues of RA patients compared to healthy individuals [68]. This aberrant activation may facilitate disease progression through multiple mechanisms. Recent studies have shown that the ANXA2/ANXA2 receptor (ANXA2R) axis promotes the secretion of downstream factors, including matrix metalloproteinase-2 (MMP-2), vascular endothelial growth factor (VEGF), and angiopoietin-2 (Ang-2), by activating the Hedgehog (HH) signaling pathway, thereby driving pannus formation [69]. Further research reveals that ANXA2 cooperatively enhances the proliferation and invasion of fibroblast-like synoviocytes (FLS) via the DDR-2/ANXA2/MMP and LncNFYB/ANXA2/ERK1/2 signaling pathways [70–71]. Additionally, ANXA2 can bind to the thrombospondin-1 (TSP1) domain of connective tissue growth factor (CTGF), forming a CTGF-ANXA2 complex that contributes to FLS proliferation, migration, and angiogenesis, ultimately facilitating pannus formation [72]. In summary, ANXA2 plays a significant role in the progression of RA.

**Table 2:** Overview of the roles of the annexin family in autoimmune diseases

**Annexin**	**Associated diseases**	**Expression change**	**Primary function**	**Related mechanisms**
ANXA1	SLE	Upregulated	Involvement in pathogenesis and progression	Modulates T-cell activation and differentiation via the ANXA1-FPR2 pathway.
	RA	Not clearly established	Dual role (Anti-inflammatory)	1. Suppresses excessive immune responses via the FPR2 pathway. 2. Inhibits cPLA2α, reducing pro-inflammatory mediators.
			Dual role (Pro-inflammatory)	The lncRNA-Anre l/miR-146a/ ANXA1 axis exacerbates inflammation.
	pSS	Upregulated	Involvement in pathogenesis	Acts synergistically with other annexin family members.
	MS	Upregulated	Protective role	Inhibits glial cell activation, maintains BBB integrity, and alleviates neuroinflammation.
ANXA2	SLE	Upregulated in LN	Key target in LN pathogenesis	Binds anti-dsDNA antibodies within the glomeruli, resulting in the formation of immune complexes that contribute to inflammation and kidney injury.
	APS	Not clearly established	Involvement in pathogenesis and progression	1. Acts as a target for anti-ANXA2, inhibiting fibrinolysis and promoting thrombosis. 2. Forms a complex with β2GPI, stimulating aβ2GPI production, activating endothelial cells, and promoting thrombosis.
	RA	Upregulated	Promotes disease progression	1. Drives pannus formation via HH pathway activation. 2. Promotes FLS proliferati on and invasion via DDR-2/ANXA2 /MMP and LncNFYB/ANX A2/ERK1/2 pathways. 3. Binds CTGF, synergistically driving disease progression.
	pSS	Upregulated	Potential biomarker	Expression level correlates with disease progression.
	MS	Upregulated	Promotes disease progression	Upregulates ICAM-1/VCAM -1, disrupts the BBB, and promotes immune cell infiltration into the CNS.
ANXA5	SLE	Not clearly established	Protective role	Binds oxCL with high affinity, mitigating endothelial dysfunction and thrombosis.
			Association with complications	aPLs disrupt its anticoagulant shield, increasing CVD risk.
	APS	Not clearly established	Association with complications	1. Disruption of its anticoagulant shield by aPLs or anti-ANXA5 increases thrombosis and/or morbid pregnancy risk. 2. anti-ANXA5 interferes with its function, promoting trophoblast apoptosis and inhibiting hCG secretion, contributing to morbid pregnancy.
	pSS	Upregulated	Involvement in pathogenesis	Acts synergistically with other annexin family members.
ANXA4, ANXA11	pSS	Upregulated	Involvement in pathogenesis	Acts synergistically with other annexin family members.

Abbreviations: Aβ2GPI: Anti-β2-glycoprotein I antibodies; aPLs: Antiphospholipid antibodies; ANXA1: Annexin A1; ANXA2: Annexin A2; ANXA4: Annexin A4; ANXA5: Annexin A5; ANXA11: Annexin A11; anti-dsDNA: Anti–double-stranded DNA antibodies; APS: Antiphospholipid syndrome; BBB: Blood–brain barrier; CNS: Central nervous system; cPLA2α: Cytosolic phospholipase A2α; CTGF: Connective tissue growth factor; CVD: Cardiovascular disease; DDR-2: Discoidin domain receptor 2; ERK1/2: Extracellular signal-regulated kinase 1/2; FLS: Fibroblast-like synoviocytes; FPR2: Formyl peptide receptor 2; hCG: Human chorionic gonadotropin; HH: Hedgehog; ICAM-1: Intercellular adhesion molecule 1; LN: Lupus nephritis; lncRNA: Long non-coding RNA; LncNFYB: Long non-coding RNA NFYB; miR-146a: MicroRNA-146a; MMP: Matrix metalloproteinase; MS: Multiple sclerosis; oxCL: Oxidized cardiolipin; pSS: Primary Sjögren’s syndrome; RA: Rheumatoid arthritis; SLE: Systemic lupus erythematosus; VCAM-1: Vascular cell adhesion molecule 1; β2GPI: β2-glycoprotein I.

## The annexin family and other autoimmune diseases

pSS is a systemic autoimmune disease primarily characterized by dysfunction of the exocrine glands, particularly the salivary and lacrimal glands, leading to severe xerostomia and keratoconjunctivitis sicca. Some patients may develop mucosa-associated lymphoid tissue lymphoma (pSS-MALT) [73–74]. Studies have shown that ANXA2 is overexpressed in the parotid glands of pSS patients, with expression levels increasing as the disease progresses from pSS to pSS-MALT [75]. Furthermore, ANXA2 has been identified as a key differentially expressed protein in salivary exosomes from pSS patients [76]. These findings suggest the potential of ANXA2 as a biomarker for diagnosing and monitoring disease activity in pSS. Notably, other annexin family members, including ANXA1, A4, A5, and A11, are also upregulated in the salivary and lacrimal glands of SS model mice, indicating that annexin family members may cooperatively participate in the pathogenesis of pSS [77].

MS is an autoimmune disease affecting the central nervous system (CNS), characterized by abnormally activated T and B cells that mediate myelin damage, leading to inflammation, demyelination, and neurodegeneration, along with blood-brain barrier (BBB) disruption and glial cell activation [78]. Recent studies suggest that ANXA1, through its anti-inflammatory properties, can inhibit glial cell activation and may play a protective role in MS by maintaining BBB integrity and mitigating neuroinflammation [79–80]. In contrast, ANXA2 exacerbates BBB disruption and promotes the infiltration of peripheral immune cells into the CNS by upregulating cell adhesion molecules ICAM-1 and VCAM-1 and regulating cytoskeletal reorganization, ultimately driving disease progression [79, 81].

## Annexin autoantibodies and autoimmune diseases

In summary, the annexin family plays significant roles in the pathogenesis of autoimmune diseases (Table 2). Moreover, as a class of emerging serological markers, their corresponding autoantibodies demonstrate multifaceted potential for application in the clinical management of these conditions (Table 3).

### Anti-ANXA1 antibodies

Anti-ANXA1 exhibits significant clinical value in the diagnosis and treatment monitoring of SLE, particularly LN. A study involving 1,052 SLE patients found that serum levels of anti-ANXA1 IgG2 were significantly higher in SLE patients with LN compared to healthy controls, correlating directly with anti-dsDNA IgG2 levels. This indicates that anti-ANXA1 IgG2 may assist in identifying early or mild LN and possesses discriminative diagnostic value [82]. Furthermore, longitudinal follow-up of newly diagnosed LN patients from disease onset to 36 months revealed that anti-ANXA1 was associated with high proteinuria, and levels of anti-ANXA1 IgG2 returned to the normal range within the first 12 months, remaining stable over the 36-month follow-up period. This suggests a relatively sensitive response of anti-ANXA1 to treatment, positioning it as a promising candidate biomarker for monitoring LN therapeutic efficacy [83]. However, this conclusion is based on a limited number of cohorts and requires further validation.

### Anti-ANXA2 antibodies

Anti-ANXA2 holds potential value in the diagnosis and risk assessment of APS. Current clinical diagnosis of APS requires at least one clinical event history plus the persistent presence of one or more autoantibodies: anticardiolipin (aCL), aβ2GPI, and lupus anticoagulant (LA). These laboratory tests should be performed concurrently and confirmed positive at least 12 weeks apart [84]. However, some patients present with typical APS clinical manifestations but test negative for these standard antiphospholipid antibodies on multiple occasions, a condition defined as seronegative APS (SNAPS) [85]. To better identify SNAPS, the additive value of “non-criteria” antibodies has gained increasing attention, including anti-phosphatidylserine/prothrombin, anti-ANXA2/ANXA5, and anti-protein S/protein C antibodies, among others [47, 86]. Although sensitivity is relatively low, studies have found that anti-ANXA2 levels are significantly higher in APS patients than in healthy individuals, providing important supplementary evidence for SNAPS diagnosis [87–89].

Moreover, clinical cohort studies have demonstrated a positive correlation between serum anti-ANXA2 levels and the incidence of thrombotic events in APS patients [89–90]. Studies in mouse models further support this association [91]. These results suggest that detecting anti-ANXA2 may assist in evaluating thrombotic risk in APS patients.

**Table 3:** Summary of the roles of annexin autoantibodies in autoimmune diseases

**Autoantibody**	**Associated disease (s)**	**Expression level**	**Clinical significance**
Anti-ANXA1 (IgG2)	SLE (especially LN)	Elevated, significantly higher in patients with LN	1. Shows potential as a biomarker for identifying early or mild LN. 2. A promising candidate biomarker for monitoring LN therapeutic efficacy; requires further validation.
Anti-ANXA2	APS	Elevated	1. Provides significant supplementary diagnostic value for SNAPS, particularly in conjunction with anti-ANXA5. 2. Association with increased thrombotic risk.
Anti-ANXA5 (IgG/IgM)	APS	Both IgG and IgM elevated	1. Provides significant supplementary diagnostic value for SNAPS, particularly in conjunction with anti-ANXA2. 2. Useful for assessing thrombosis risk. 3. The association with morbid pregnancy remains controversial.
	SSc	Elevated (IgG)	1. Positivity is associated with greater disease severity (digital vasculopathy, lung fibrosis), useful for severity assessment. 2. Potential value for long-term disease monitoring.

Abbreviations: ANXA1: Annexin A1; ANXA2: Annexin A2; ANXA5: Annexin A5; APS: Antiphospholipid syndrome; LN: Lupus nephritis; SLE: Systemic lupus erythematosus; SNAPS: Seronegative antiphospholipid syndrome; SSc: Systemic sclerosis.

### Anti-ANXA5 antibodies

The clinical significance of anti-ANXA5 antibodies has been established in both SSc and APS. SSc is a rare autoimmune connective tissue disorder characterized by skin and internal organ fibrosis, as well as vasculopathy [92]. Studies have shown that serum levels of anti-ANXA5 are significantly elevated in SSc patients compared to healthy controls, and positivity for anti-ANXA5 IgG correlates with increased severity of digital vasculopathy and lung fibrosis [93–95]. Notably, anti-ANXA5 can remain consistently positive for up to two years, indicating its potential utility for long-term disease monitoring [96].

In the context of APS, anti-ANXA5 antibodies are crucial for disease diagnosis and risk assessment. Clinical evaluations have revealed that some patients with SNAPS are persistently positive for anti-ANXA5 IgG/M [97–98]. A cohort study conducted in China demonstrated elevated levels of both anti-ANXA5 IgG and IgM in APS patients, and statistical analyses indicated that the detection of anti-ANXA5, particularly anti-ANXA5 IgG, could enhance the diagnostic sensitivity for APS [99]. These findings suggest that anti-ANXA5 testing can improve clinical diagnostic performance for APS, providing critical supplemental value for SNAPS. A clinical study involving 70 patients indicated that anti-ANXA5 not only aids in APS diagnosis but can also be employed to assess thrombotic risk [100]. Furthermore, research utilizing a modified thrombin generation assay found that 40.7% of anti-ANXA5 positive APS patients reached a laboratory thrombogenicity threshold (AUC R ≤ 4.5), a significantly higher proportion than that of anti-ANXA5 negative patients [101], further underscoring the role of anti-ANXA5 in assessing thrombosis risk. Regarding obstetric complications, while multiple studies have reported a significant association between anti-ANXA5 positivity and risks such as preterm birth and recurrent spontaneous abortion (RSA) in APS patients [25, 102–105], some studies have failed to confirm this association [99, 106–108]. This discrepancy highlights the necessity for further research to elucidate the specific mechanisms and clinical implications of anti-ANXA5 in obstetric APS.

**Figure 1.**
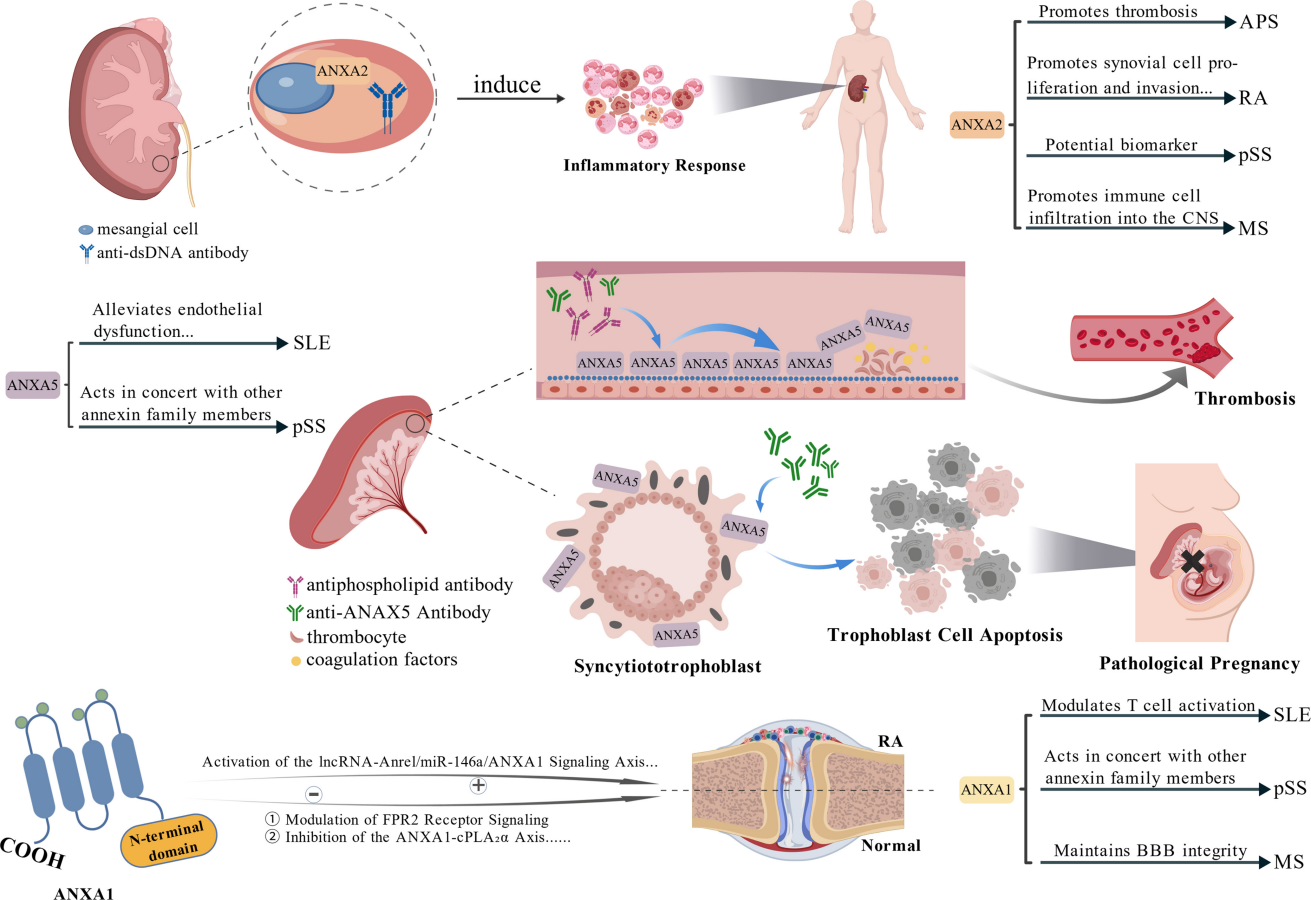
**The roles of the annexin family in autoimmune diseases.** Schematic summary of disease-associated functions of ANXA2, ANXA5, and ANXA1. ANXA2 is depicted binding nephritogenic anti-dsDNA on mesangial cells to induce an inflammatory response, with indicated links to thrombosis in APS, synovial proliferation/invasion in RA, biomarker potential in pSS, and immune-cell infiltration into the CNS in MS. ANXA5 is shown forming an endothelial/placental anticoagulant “shield” that is disrupted by antiphospholipid antibodies and anti-ANXA5, promoting thrombosis and trophoblast apoptosis leading to pathological pregnancy. ANXA1-related signaling and downstream effects are illustrated alongside its reported roles in SLE, pSS, and maintenance of BBB integrity in MS. *This figure was generated using BioGDP.com*^[109]^. Abbreviations: ANXA1: Annexin A1; ANXA2: Annexin A2; ANXA5: Annexin A5; anti-dsDNA: Anti–double-stranded DNA antibodies; APS: Antiphospholipid syndrome; BBB: Blood–brain barrier; CNS: Central nervous system; MS: Multiple sclerosis; pSS: Primary Sjögren’s syndrome; RA: Rheumatoid arthritis; SLE: Systemic lupus erythematosus.

## Conclusion and perspectives

Annexins and their autoantibodies play critical roles in various autoimmune diseases (Figure 1) [109]. Based on the current evidence synthesized in this review, ANXA1, A2, and A5 exhibit particularly significant regulatory functions in the discussed pathologies. It is important to note that other members of the annexin family (e.g., ANXA4, ANXA11) may also contribute to regulatory and pathophysiological processes in autoimmunity; however, existing data on these proteins remain insufficient and warrant further investigation. In SLE/LN, ANXA1, ANXA2, and ANXA5 function synergistically, accompanied by a marked increase in anti-ANXA1. APS is primarily characterized by dysregulation of ANXA2 and ANXA5, closely associated with the pathogenic roles of anti-ANXA2 and anti-ANXA5. The synovial pathology in RA involves the dual regulatory function of ANXA1 and ANXA2-mediated processes of synovial hyperplasia and invasion. pSS exhibits upregulation of multiple annexin family members, including ANXA1, A2, A4, A5, and A11, in salivary glands. In MS, ANXA1 provides a protective role at the blood-brain barrier, while ANXA2 contributes to barrier disruption and disease progression. Although current research has begun to elucidate the mechanisms of the annexin family in autoimmune diseases, significant limitations persist: (1) most clinical data stem from single-center, small-sample cohorts, and inconsistencies in detection methods and positivity criteria hinder the comparability and generalizability of results; (2) study designs are predominantly cross-sectional, lacking prospective, multicenter, long-term follow-up data to validate their clinical translational value. Future studies should focus on: (1) further elucidating the molecular mechanisms of annexin family members in various diseases and developing targeted therapeutic strategies against specific annexin pathways; (2) establishing standardized detection systems and conducting multicenter, prospective clinical studies to systematically validate the clinical applicability of annexin-related antibodies (e.g., anti-ANXA2, anti-ANXA5) as diagnostic and prognostic biomarkers; (3) conducting in-depth research on the interactions between annexins and other biomolecules, as well as inflammatory pathways, to provide a theoretical basis for identifying new disease classification markers and therapeutic targets. The diverse functions of the annexin family in autoimmune diseases position them as promising candidates for future precision medicine research. Integrating clinical and basic research holds the potential for their translational application in disease prediction, subtyping, and intervention.

## Data Availability

Data sharing is not applicable to this article as no new data were generated or analyzed in this study.

## References

[1] Conrad N, Misra S, Verbakel JY, Verbeke G, Molenberghs G, Taylor PN (2023). Incidence, prevalence, and co-occurrence of autoimmune disorders over time and by age, sex, and socioeconomic status: a population-based cohort study of 22 million individuals in the UK. Lancet.

[2] Luo B, Xiang D, Ji X, Chen X, Li R, Zhang S (2024). The anti-inflammatory effects of exercise on autoimmune diseases: a 20-year systematic review. J Sport Health Sci.

[3] Miller FW (2023). The increasing prevalence of autoimmunity and autoimmune diseases: an urgent call to action for improved understanding, diagnosis, treatment, and prevention. Curr Opin Immunol.

[4] Gerke V, Gavins FNE, Geisow M, Grewal T, Jaiswal JK, Nylandsted J (2024). Annexins–a family of proteins with distinctive tastes for cell signaling and membrane dynamics. Nat Commun.

[5] Qian Z, Li Z, Peng X, Mao Y, Mao X, Li J (2025). Annexin A: Cell death, inflammation, and translational medicine. J Inflamm Res.

[6] Huang Y, Jia M, Yang X, Han H, Hou G, Bi L (2022). Annexin A2:The diversity of pathological effects in tumorigenesis and immune response. Int J Cancer.

[7] Zhang H, Zhang Z, Guo T, Chen G, Liu G, Song Q (2023). Annexin A protein family: focusing on the occurrence, progression and treatment of cancer. Front Cell Dev Biol.

[8] Hu J, Chen L, Ruan J, Chen X (2024). The role of the annexin A protein family at the maternal-fetal interface. Front Endocrinol (Lausanne).

[9] Fang L, Liu C, Jiang ZZ, Wang M, Geng K, Xu Y (2024). Annexin A1 binds PDZ and LIM domain 7 to inhibit adipogenesis and prevent obesity. Signal Transduct Target Ther.

[10] Pan H, Guo Z, Lv P, Hu K, Wu T, Lin Z (2023). Proline/serine-rich coiled-coil protein 1 inhibits macrophage inflammation and delays atherosclerotic progression by binding to Annexin A2. Clin Transl Med.

[11] Gerke V, Moss SE (2002). Annexins: from structure to function. Physiol Rev.

[12] Mirsaeidi M, Gidfar S, Vu A, Schraufnagel D (2016). Annexins family: insights into their functions and potential role in pathogenesis of sarcoidosis. J Transl Med.

[13] Grewal T, Rentero C, Enrich C, Wahba M, Raabe CA, Rescher U (2021). Annexin animal models–from fundamental principles to translational research. Int J Mol Sci.

[14] Araújo TG, Mota STS, Ferreira HSV, Ribeiro MA, Goulart LR, Vecchi L (2021). Annexin A1 as a regulator of immune response in cancer. Cells.

[15] Sousa SO, Santos MRD, Teixeira SC, Ferro EAV, Oliani SM (2022). Annexin A1: roles in placenta, cell survival, and nucleus. Cells.

[16] Xu X, Gao W, Li L, Hao J, Yang B, Wang T (2021). Annexin A1 protects against cerebral ischemia-reperfusion injury by modulating microglia/macrophage polarization via FPR2/ALX-dependent AMPK-mTOR pathway. J Neuroinflammation.

[17] You Q, Ke Y, Chen X, Yan W, Li D, Chen L (2024). Loss of endothelial annexin A1 aggravates inflammation-induced vascular aging. Adv Sci (Weinh).

[18] Li C, Yu J, Liao D, Su X, Yi X, Yang X (2023). Annexin A2: the missing piece in the puzzle of pathogen-induced damage. Virulence.

[19] Wang T, Zhao D, Zhang Y, Yu D, Liu G, Zhang K (2024). Annexin A2: a double-edged sword in pathogen infection. Pathogens.

[20] Christofidis K, Pergaris A, Fioretzaki R, Charalampakis N, Kapetanakis EI, Kavantzas N (2024). Annexin A2 in tumors of the gastrointestinal tract, liver, and pancreas. Cancers (Basel).

[21] Luo M, Almeida D, Dallacasagrande V, Hedhli N, Gupta M, D’Amico DJ (2024). Annexin A2 promotes proliferative vitreoretinopathy in response to a macrophage inflammatory signal in mice. Nat Commun.

[22] Boersma HH, Kietselaer BL, Stolk LM, Bennaghmouch A, Hofstra L, Narula J (2005). Past, present, and future of annexin A5: from protein discovery to clinical applications. J Nucl Med.

[23] Jin M, Zhang J, Sun Y, Liu G, Wei X (2025). ANXA5: related mechanisms of osteogenesis and additional biological functions. Front Cell Dev Biol.

[24] Jing J (2024). The relevance, predictability, and utility of annexin A5 for human physiopathology. Int J Mol Sci.

[25] Murad H, Ali B, Twair A, Baghdadi K, Alhalabi M, Abbady AQ (2023). “In House” assays for the quantification of Annexin V and its autoantibodies in patients with recurrent pregnancy loss and in vitro fertilisation failures. Sci Rep.

[26] Siegel CH, Sammaritano LR (2024). Systemic lupus erythematosus: a review. JAMA.

[27] Tian J, Zhang D, Yao X, Huang Y, Lu Q (2023). Global epidemiology of systemic lupus erythematosus: a comprehensive systematic analysis and modelling study. Ann Rheum Dis.

[28] Ameer MA, Chaudhry H, Mushtaq J, Khan OS, Babar M, Hashim T (2022). An overview of systemic lupus erythematosus (SLE) pathogenesis, classification, and management. Cureus.

[29] Su X, Yu H, Lei Q, Chen X, Tong Y, Zhang Z (2024). Systemic lupus erythematosus: pathogenesis and targeted therapy. Mol Biomed.

[30] Perretti M, D’Acquisto F (2009). Annexin A1 and glucocorticoids as effectors of the resolution of inflammation. Nat Rev Immunol.

[31] Li Y, Xu B, Zhang J, Liu X, Ganesan K, Shi G (2023). Exploring the role of LIAS-related cuproptosis in systemic lupus erythematosus. Lupus.

[32] Mihaylova N, Chipinski P, Bradyanova S, Velikova T, Ivanova-Todorova E, Chausheva S (2020). Suppression of autoreactive T and B lymphocytes by anti-annexin A1 antibody in a humanized NSG murine model of systemic lupus erythematosus. Clin Exp Immunol.

[33] Dhaffouli F, Hachicha H, Abida O, Gharbi N, Elloumi N, Kanoun H (2022). Annexin A1 and its receptor gene polymorphisms in systemic lupus erythematosus in the Tunisian population. Clin Rheumatol.

[34] Parikh SV, Almaani S, Brodsky S, Rovin BH (2020). Update on lupus nephritis: Core Curriculum 2020. Am J Kidney Dis.

[35] Hoi A, Igel T, Mok CC, Arnaud L (2024). Systemic lupus erythematosus. Lancet.

[36] Roveta A, Parodi EL, Brezzi B, Tunesi F, Zanetti V, Merlotti G (2024). Lupus nephritis from pathogenesis to new therapies: an update. Int J Mol Sci.

[37] Tsai CY, Li KJ, Shen CY, Lu CH, Lee HT, Wu TH (2023). Decipher the immunopathological mechanisms and set up potential therapeutic strategies for patients with lupus nephritis. Int J Mol Sci.

[38] Yung S, Cheung KF, Zhang Q, Chan TM (2010). Anti-dsDNA antibodies bind to mesangial annexin II in lupus nephritis. J Am Soc Nephrol.

[39] Dörner T, Furie R (2019). Novel paradigms in systemic lupus erythematosus. Lancet.

[40] Tektonidou MG, Lewandowski LB, Hu J, Dasgupta A, Ward MM (2017). Survival in adults and children with systemic lupus erythematosus: a systematic review and Bayesian meta-analysis of studies from 1950 to 2016. Ann Rheum Dis.

[41] Frostegård J (2023). Systemic lupus erythematosus and cardiovascular disease. J Intern Med.

[42] Ruiz-Irastorza G, Crowther M, Branch W, Khamashta MA (2010). Antiphospholipid syndrome. Lancet.

[43] Cederholm A, Svenungsson E, Jensen-Urstad K, Trollmo C, Ulfgren AK, Swedenborg J (2005). Decreased binding of annexin V to endothelial cells: a potential mechanism in atherothrombosis of patients with systemic lupus erythematosus. Arterioscler Thromb Vasc Biol.

[44] Su J, Frostegård AG, Hua X, Gustafsson T, Jogestrand T, Hafström I (2013). Low levels of antibodies against oxidized but not nonoxidized cardiolipin and phosphatidylserine are associated with atherosclerotic plaques in systemic lupus erythematosus. J Rheumatol.

[45] Cederholm A, Frostegård J (2007). Annexin A5 as a novel player in prevention of atherothrombosis in SLE and in the general population. Ann N Y Acad Sci.

[46] Wan M, Hua X, Su J, Thiagarajan D, Frostegård AG, Haeggström JZ (2014). Oxidized but not native cardiolipin has pro-inflammatory effects, which are inhibited by Annexin A5. Atherosclerosis.

[47] Knight JS, Branch DW, Ortel TL (2023). Antiphospholipid syndrome: advances in diagnosis, pathogenesis, and management. BMJ.

[48] Duarte-García A, Pham MM, Crowson CS, Amin S, Moder KG, Pruthi RK (2019). The epidemiology of antiphospholipid syndrome: a population-based study. Arthritis Rheumatol.

[49] Dabit JY, Valenzuela-Almada MO, Vallejo-Ramos S, Duarte-García A (2022). Epidemiology of antiphospholipid syndrome in the general population. Curr Rheumatol Rep.

[50] Brownstein C, Deora AB, Jacovina AT, Weintraub R, Gertler M, Khan KM (2004). Annexin II mediates plasminogen-dependent matrix invasion by human monocytes: enhanced expression by macrophages. Blood.

[51] Cesarman-Maus G, Cantú-Brito C, Barinagarrementeria F, Villa R, Reyes E, Sanchez-Guerrero J (2011). Autoantibodies against the fibrinolytic receptor, annexin A2, in cerebral venous thrombosis. Stroke.

[52] Green D (2022). Pathophysiology of antiphospholipid syndrome. Thromb Haemost.

[53] Antovic A, Bruzelius M (2021). Impaired fibrinolysis in the antiphospholipid syndrome. Semin Thromb Hemost.

[54] Zhang J, McCrae KR (2005). Annexin A2 mediates endothelial cell activation by antiphospholipid/anti-beta2 glycoprotein I antibodies. Blood.

[55] Rand JH (2000). Antiphospholipid antibody-mediated disruption of the annexin-V antithrombotic shield: a thrombogenic mechanism for the antiphospholipid syndrome. J Autoimmun.

[56] Roselli D, Bonifacio MA, Barbuti G, Rossiello MR, Ranieri P, Mariggiò MA (2023). Anti-phosphatidylserine, anti-prothrombin, and anti-annexin V autoantibodies in antiphospholipid syndrome: a real-life study. Diagnostics (Basel).

[57] Alijotas-Reig J, Esteve-Valverde E, Anunciación-Llunell A, Marques-Soares J, Pardos-Gea J, Miró-Mur F (2022). Pathogenesis, diagnosis and management of obstetric antiphospholipid syndrome: a comprehensive review. J Clin Med.

[58] Mekinian A, Alijotas-Reig J, Carrat F, Costedoat-Chalumeau N, Ruffatti A, Lazzaroni MG (2017). Refractory obstetrical antiphospholipid syndrome: features, treatment and outcome in a European multicenter retrospective study. Autoimmun Rev.

[59] Di Simone N, Castellani R, Caliandro D, Caruso A (2001). Monoclonal anti-annexin V antibody inhibits trophoblast gonadotropin secretion and induces syncytiotrophoblast apoptosis. Biol Reprod.

[60] Di Matteo A, Bathon JM, Emery P (2023). Rheumatoid arthritis. Lancet.

[61] Gao Y, Zhang Y, Liu X (2024). Rheumatoid arthritis: pathogenesis and therapeutic advances. MedComm (2020).

[62] Ding Q, Hu W, Wang R, Yang Q, Zhu M, Li M (2023). Signaling pathways in rheumatoid arthritis: implications for targeted therapy. Signal Transduct Target Ther.

[63] Yang YH, Morand E, Leech M (2013). Annexin A1: potential for glucocorticoid sparing in RA. Nat Rev Rheumatol.

[64] Solito E, de Coupade C, Parente L, Flower RJ, Russo-Marie F (1998). IL-6 stimulates annexin 1 expression and translocation and suggests a new biological role as class II acute phase protein. Cytokine.

[65] Yanding G, Kun L, Linlin Z, Wenting LU, Yanan S, Yumei Z (2024). Study on the anti-inflammatory mechanism of moxibustion in rheumatoid arthritis in rats based on phospholipaseA2 signaling inhibition by Annexin 1. J Tradit Chin Med.

[66] Chen J, Norling LV, Mesa JG, Silva MP, Burton SE, Reutelingsperger C (2021). Annexin A1 attenuates cardiac diastolic dysfunction in mice with inflammatory arthritis. Proc Natl Acad Sci U S A.

[67] Wang J, Zhao J, Lin L, Peng X, Li W, Huang Y (2023). LncRNA-Anrel promotes the proliferation and migration of synovial fibroblasts through regulating miR- 146a-mediated annexin A1 expression. Am J Clin Exp Immunol.

[68] Tsen SD, Springer LE, Sharmah Gautam K, Tang R, Liang K, Sudlow G (2021). Non-invasive monitoring of arthritis treatment response via targeting of tyrosine-phosphorylated annexin A2 in chondrocytes. Arthritis Res Ther.

[69] Yi J, Zhu Y, Jia Y, Jiang H, Zheng X, Liu D (2016). The Annexin A2 promotes development in arthritis through neovascularization by amplification Hedgehog pathway. PLoS One.

[70] Xiao S, Ouyang Q, Feng Y, Lu X, Han Y, Ren H (2024). LncNFYB promotes the proliferation of rheumatoid arthritis fibroblast-like synoviocytes via LncNFYB/ANXA2/ERK1/2 axis. J Biol Chem.

[71] Zhao W, Zhang C, Shi M, Zhang J, Li M, Xue X (2014). The discoidin domain receptor 2/annexin A2/matrix metalloproteinase 13 loop promotes joint destruction in arthritis through promoting migration and invasion of fibroblast-like synoviocytes. Arthritis Rheumatol.

[72] Yin G, Yang C, Wu G, Yu X, Tian Q, Chen D (2021). The protein-protein interaction between connective tissue growth factor and annexin A2 is relevant to pannus formation in rheumatoid arthritis. Arthritis Res Ther.

[73] Baldini C, Fulvio G, La Rocca G, Ferro F (2024). Update on the pathophysiology and treatment of primary Sjögren syndrome. Nat Rev Rheumatol.

[74] Liang Y, Yang Z, Qin B, Zhong R (2014). Primary Sjogren’s syndrome and malignancy risk: a systematic review and meta-analysis. Ann Rheum Dis.

[75] Cui L, Elzakra N, Xu S, Xiao GG, Yang Y, Hu S (2017). Investigation of three potential autoantibodies in Sjogren’s syndrome and associated MALT lymphoma. Oncotarget.

[76] Finamore F, Cecchettini A, Ceccherini E, Signore G, Ferro F, Rocchiccioli S (2021). Characterization of extracellular vesicle cargo in Sjögren’s syndrome through a SWATH-MS proteomics approach. Int J Mol Sci.

[77] Peck AB, Ambrus JL Jr (2022). A temporal comparative RNA transcriptome profile of the annexin gene family in the salivary versus lacrimal glands of the Sjögren’s syndrome-susceptible C57BL/6.NOD-Aec1Aec2 mouse. Int J Mol Sci.

[78] McGinley MP, Goldschmidt CH, Rae-Grant AD (2021). Diagnosis and treatment of multiple sclerosis: a review. JAMA.

[79] White ZB 2nd, Nair S, Bredel M (2024). The role of annexins in central nervous system development and disease. J Mol Med (Berl).

[80] Hejazi MS, Jafari S, Montazersaheb S, Molavi O, Hosseini V, Talebi M (2024). Annexin A1, calreticulin and high mobility group box 1 are elevated in secondary progressive multiple sclerosis: does immunogenic cell death occur in multiple sclerosis?. Bioimpacts.

[81] Tezuka K, Suzuki M, Sato R, Kawarada S, Terasaki T, Uchida Y (2022). Activation of annexin A2 signaling at the blood-brain barrier in a mouse model of multiple sclerosis. J Neurochem.

[82] Bruschi M, Moroni G, Sinico RA, Franceschini F, Fredi M, Vaglio A (2021). Serum IgG2 antibody multicomposition in systemic lupus erythematosus and lupus nephritis (Part 1): cross-sectional analysis. Rheumatology (Oxford).

[83] Bruschi M, Moroni G, Sinico RA, Franceschini F, Fredi M, Vaglio A (2021). Serum IgG2 antibody multi-composition in systemic lupus erythematosus and in lupus nephritis (Part 2): prospective study. Rheumatology (Oxford).

[84] Miyakis S, Lockshin MD, Atsumi T, Branch DW, Brey RL, Cervera R (2006). International consensus statement on an update of the classification criteria for definite antiphospholipid syndrome (APS). J Thromb Haemost.

[85] Jawad AS (2004). Seronegative antiphospholipid syndrome. Ann Rheum Dis.

[86] Sciascia S, Amigo MC, Roccatello D (2017). Diagnosing antiphospholipid syndrome: “extra-criteria” manifestations and technical advances. Nat Rev Rheumatol.

[87] Bradacova P, Slavik L, Ulehlova J, Skoumalova A, Ullrychova J, Prochazkova J (2021). Current promising biomarkers and methods in the diagnostics of antiphospholipid syndrome: a review. Biomedicines.

[88] Cañas F, Simonin L, Couturaud F, Renaudineau Y (2015). Annexin A2 autoantibodies in thrombosis and autoimmune diseases. Thromb Res.

[89] Cesarman-Maus G, Ríos-Luna NP, Deora AB, Huang B, Villa R, Cravioto Mdel C (2006). Autoantibodies against the fibrinolytic receptor, annexin 2, in antiphospholipid syndrome. Blood.

[90] Ao W, Zheng H, Chen XW, Shen Y, Yang CD (2011). Anti-annexin II antibody is associated with thrombosis and/or pregnancy morbidity in antiphospholipid syndrome and systemic lupus erythematosus with thrombosis. Rheumatol Int.

[91] Weiss R, Bushi D, Mindel E, Bitton A, Diesendruck Y, Gera O (2021). Autoantibodies to Annexin A2 and cerebral thrombosis: insights from a mouse model. Lupus.

[92] Volkmann ER, Andréasson K, Smith V (2023). Systemic sclerosis. Lancet.

[93] Sugiura K, Muro Y (1999). Anti-annexin V antibodies and digital ischemia in patients with scleroderma. J Rheumatol.

[94] Esposito G, Tamby MC, Chanseaud Y, Servettaz A, Guillevin L, Mouthon L (2005). Anti-annexin V antibodies: are they prothrombotic?. Autoimmun Rev.

[95] El Serougy IM, Shahin AA, Soliman DA, Akhnoukh AF, Mousa SM (2009). Clinical significance of serum anti-annexin V antibodies in Egyptian patients with scleroderma. Egypt J Immunol.

[96] Horimoto AMC, de Jesus LG, de Souza AS, Rodrigues SH, Kayser C (2020). Anti-annexin V autoantibodies and vascular abnormalities in systemic sclerosis: a longitudinal study. Adv Rheumatol.

[97] Conti F, Andreoli L, Crisafulli F, Mancuso S, Truglia S, Tektonidou MG (2019). Does seronegative obstetric APS exist? “pro” and “cons”. Autoimmun Rev.

[98] Ortona E, Capozzi A, Colasanti T, Conti F, Alessandri C, Longo A (2010). Vimentin/cardiolipin complex as a new antigenic target of the antiphospholipid syndrome. Blood.

[99] Zhang S, Wu Z, Li J, Wen X, Li L, Zhang W (2017). Evaluation of the clinical relevance of anti-annexin-A5 antibodies in Chinese patients with antiphospholipid syndrome. Clin Rheumatol.

[100] Lakos G, Kiss E, Regeczy N, Tarjan P, Soltesz P, Zeher M (2000). Antiprothrombin and antiannexin V antibodies imply risk of thrombosis in patients with systemic autoimmune diseases. J Rheumatol.

[101] Bradáčová P, Slavík L, Skoumalová A, Úlehlová J, Kriegová E, Manukyan G (2022). Determination of thrombogenicity levels of various antiphospholipid antibodies by a modified thrombin generation assay in patients with suspected antiphospholipid syndrome. Int J Mol Sci.

[102] Matsubayashi H, Arai T, Izumi S, Sugi T, McIntyre JA, Makino T (2001). Anti-annexin V antibodies in patients with early pregnancy loss or implantation failures. Fertil Steril.

[103] Xiong Y, Wu T, Wang L, Shen X, Yin Y, Wu J (2025). The clinical significance of non-criteria antiphospholipid antibodies in atypical antiphospholipid syndrome. Mod Rheumatol.

[104] Gris JC, Quéré I, Sanmarco M, Boutiere B, Mercier E, Amiral J (2000). Antiphospholipid and antiprotein syndromes in non-thrombotic, non-autoimmune women with unexplained recurrent primary early foetal loss. The Nîmes Obstetricians and Haematologists Study–NOHA. Thromb Haemost.

[105] Guo X, Xiang J, Zhang W, Zheng X, Dai Y, Cai Z (2024). Association of anti-annexin A5 antibody with pregnancy outcomes: a cohort study: Anti-annexin A5 antibody with pregnancy outcomes. Am J Reprod Immunol.

[106] Nasef A, Ibrahim M, Riad N, Mousa S (2014). Plasma annexin A5, anti-annexin A5 antibodies and annexin A5 polymorphism in Egyptian female patients with systemic lupus erythematosus and antiphospholipid syndrome. Clin Lab.

[107] Alijotas-Reig J, Esteve-Valverde E, Ferrer-Oliveras R, Sáez-Comet L, Lefkou E, Mekinian A (2019). The European Registry on Obstetric Antiphospholipid Syndrome (EUROAPS): a survey of 1000 consecutive cases. Autoimmun Rev.

[108] Bećarević M (2016). The IgG and IgM isotypes of anti-annexin A5 antibodies: relevance for primary antiphospholipid syndrome. J Thromb Thrombolysis.

[109] Jiang S, Li H, Zhang L, Mu W, Zhang Y, Chen T (2025). Generic Diagramming Platform (GDP): a comprehensive database of high-quality biomedical graphics. Nucleic Acids Res.

